# 3-Iodo­aniline

**DOI:** 10.1107/S2414314624012264

**Published:** 2024-12-24

**Authors:** Ferhan Slamang, Eric Cyriel Hosten, Richard Betz

**Affiliations:** a Nelson Mandela University, Summerstrand Campus, Department of Chemistry, University Way, Summerstrand, PO Box 77000, Port Elizabeth, 6031, South Africa; University of Antofagasta, Chile

**Keywords:** crystal structure

## Abstract

The title compound, C_6_H_6_IN, is the *meta*-iodinated derivative of aniline. The asymmetric unit contains two mol­ecules. The structure was refined as a two-component inversion twin with a volume ratio of 55.6:44.4. In the crystal, cooperative hydrogen bonds of the N–H⋯N type connect the mol­ecules into infinite chains propagating along the *a-*axis direction. Dispersive I⋯I contacts extend these chains to sheets perpendicular to the crystallographic *c* axis.

## Abstract 2

The asymmetric unit of the title compound, the *meta*-iodinated derivative of aniline, contains two complete mol­ecules. In the crystal, cooperative hydrogen bonds of the N–H⋯N type connect the mol­ecules into infinite chains along the *a-*axis direction. Dispersive I⋯I contacts extend these chains to sheets perpendicular to the crystallographic *c* axis.

## Graphical abstract

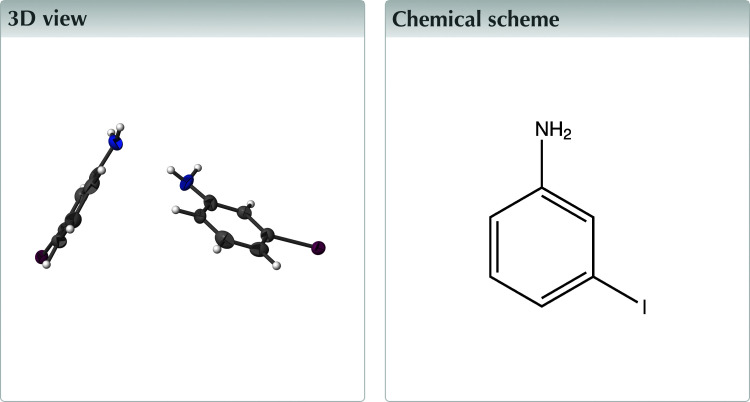

## Structure description

Aniline and its derivatives are valuable starting materials in synthetic organic chemistry and have found ample use in industrial processes, as is apparent in the historic establishment of the artificial dye and, subsequently, pharmaceutical industry (Griess, 1879 ▸; Bopp *et al.*, 1891 ▸). As an activated aromatic system, a large number of reactions is available for further functionalization of the phenyl group as well as the *ipso*-substitution of the amine functionality itself (Becker *et al.*, 2000 ▸; Sandmeyer, 1884 ▸), which allows for tailoring the physicochemical and spectroscopic properties of the target mol­ecules over a seemingly endless range. In a continuation of our own inter­est in the structural variety of aniline derivatives (Islor *et al.*, 2013 ▸; Betz & Gerber, 2011 ▸; Betz *et al.*, 2008 ▸, 2011*a* ▸,*b* ▸; Betz, 2015 ▸; Hosten & Betz, 2021*a* ▸,*b* ▸,*c* ▸) as well as pyridine-based amines (Betz *et al.*, 2011*c* ▸,*d* ▸) we sought to determine the structure of 3-iodo­aniline. Structural information about this mol­ecule is scarce as only protonated versions of the compound under investigation are apparent in the literature, such as the chloride (Xing *et al.*, 2021 ▸), iodide (Gray & Jones, 2002 ▸), phosphate (Yoshii *et al.*, 2015 ▸), *ortho*-nitro­phthalate (Glidewell *et al.*, 2005 ▸) as well as the crown-ether-supported salts of an anionic nickel coordination compound (Kubo *et al.*, 2021 ▸) and two Keggin-ion-inspired polyoxometallates derived from molybdenum (Xiong *et al.*, 2015 ▸, 2016 ▸), with a metal–organic molybdenum coordination compound being the only example in which structural data about the neutral title compound – as a ligand – is available (Xing *et al.*, 2021 ▸).

**Figure 1:**
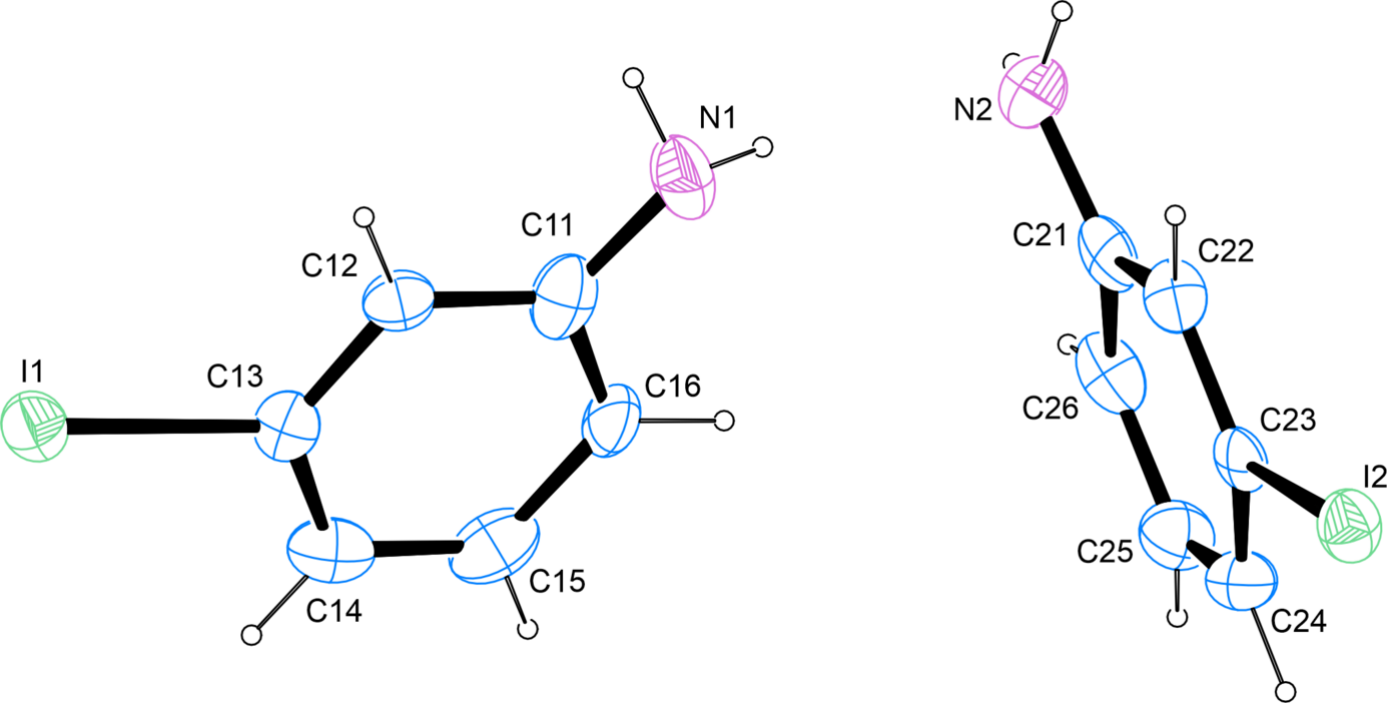
The mol­ecular structure of the title compound, with atom labels and anisotropic displacement ellipsoids (drawn at the 50% probability level).

The title compound is the *meta*-iodinated derivative of aniline. The asymmetric unit contains two mol­ecules. The structure was refined as an inversion twin with a volume ratio of 55.6:44.4. The C—I bond lengths of 2.105 (10) and 2.113 (11) Å are in good agreement with other aromatic iodine compounds whose metrical parameters have been determined on the basis of diffraction studies on single crystals and whose metrical parameters have been deposited with the Cambridge Structural Database (Groom *et al.*, 2016 ▸). The intra­cyclic C—C—C angles span a range of 117.9 (11)–122.4 (11)° in the first and 117.9 (9)–122.5 (10)° in the second mol­ecule present in the asymmetric unit with the smallest angle on the carbon atom in *para*-position to the amino group in the first mol­ecule and in *ortho*-position to the amino group in the second mol­ecule. The largest C—C—C angle is invariably found on the carbon atom bearing the halogen substituent. The least-squares planes as defined by the carbon atoms of the two respective aromatic systems enclose an angle of 83.3 (5)° (Fig. 1 ▸).

**Figure 2:**
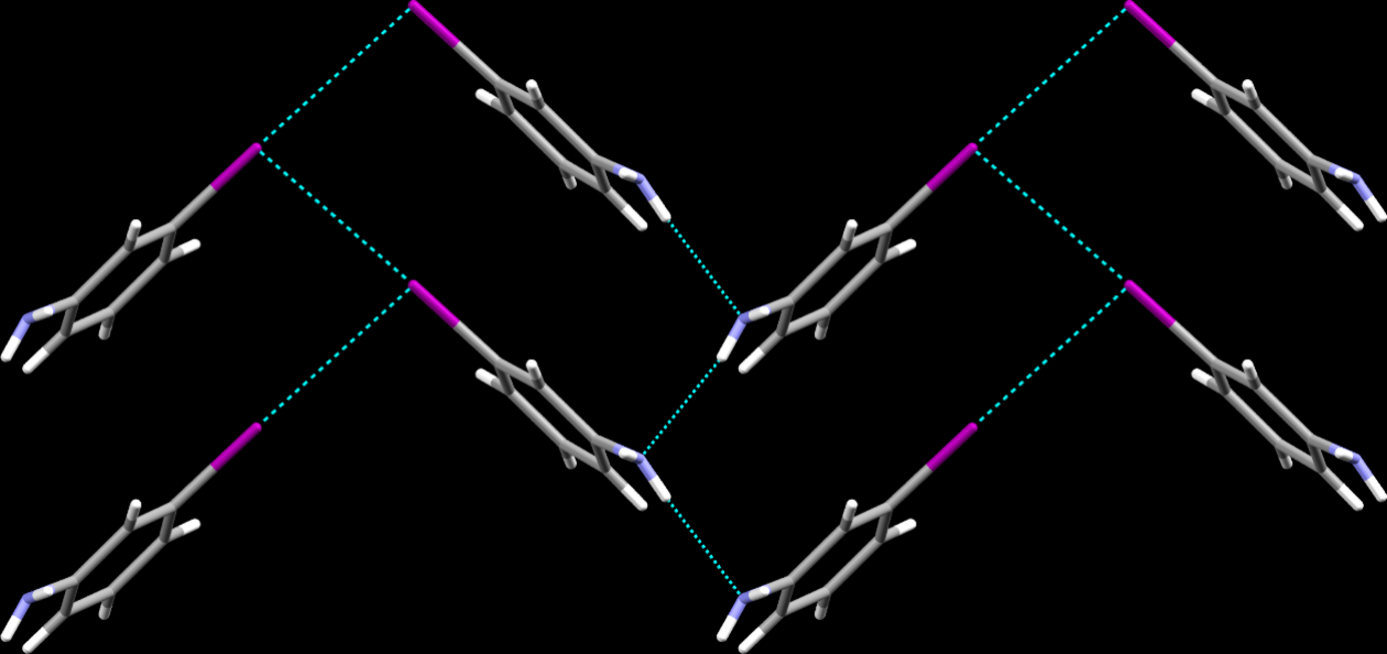
Inter­molecular contacts, viewed along [001].

In the crystal, cooperative hydrogen bonds of the N—H⋯N type (Table 1 ▸) are apparent that are supported by only one hydrogen in each amino group. These connect the mol­ecules into infinite chains propagating along the *a-*axis direction. In terms of graph-set analysis (Etter *et al.*, 1990 ▸; Bernstein *et al.*, 1995 ▸), the descriptor for these hydrogen bonds is *DD* on the unary level. Furthermore, dispersive I⋯I contacts are observed whose range of 3.79 (1)–3.85 (1) Å falls by more than 0.1 Å below the sum of the van der Waals radii of the atoms participating in them. The latter extend the chains to sheets lying perpendicular to the crystallographic *c* axis. π-Stacking is not a prominent stabilizing feature in the crystal structure of the title compound with the shortest inter­centroid distance measured at 5.074 (6) Å between the aromatic system of one of the two mol­ecules present in the asymmetric unit and its symmetry-generated equivalent, which corresponds to the *b*-axis unit-cell dimension (Fig. 2 ▸).

## Synthesis and crystallization

The title compound was obtained commercially (Sigma-Aldrich). A crystal suitable for the diffraction study was obtained upon prolonged and repeated sublimation and re-sublimation of the compound at a temperature just above 0°C in a fridge.

## Refinement

Crystal data, data collection and structure refinement details are summarized in Table 2 ▸.

## Supplementary Material

Crystal structure: contains datablock(s) I. DOI: 10.1107/S2414314624012264/bx4032sup1.cif

Structure factors: contains datablock(s) I. DOI: 10.1107/S2414314624012264/bx4032Isup2.hkl

Supporting information file. DOI: 10.1107/S2414314624012264/bx4032Isup3.cml

CCDC reference: 2411477

Additional supporting information:  crystallographic information; 3D view; checkCIF report

## Figures and Tables

**Table 1:** Hydrogen-bond geometry (Å, °)

*D*—H⋯*A*	*D*—H	H⋯*A*	*D*⋯*A*	*D*—H⋯*A*
N1—H711⋯N2	0.78 (15)	2.38 (16)	3.156 (17)	170 (15)
N2—H722⋯N1^i^	0.84 (15)	2.33 (15)	3.157 (18)	171 (13)
C13—I1⋯I2^ii^	2.11 (1)	3.85 (1)	8.677 (10)	173 (1)
C23—I2⋯I1^iii^	2.11 (1)	3.79 (1)	9.437 (9)	172 (1)

Symmetry codes: (i) 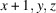; (ii) 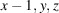; (iii) 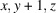.

**Table 2:** Experimental details

Crystal data	
Chemical formula	C_6_H_6_IN
*M*_r_	219.02
Crystal system, space group	Orthorhombic, *P*2_1_2_1_2_1_
Temperature (K)	200
*a*, *b*, *c* (Å)	5.0748 (3), 12.9872 (8), 20.6243 (12)
*V* (Å^3^)	1359.29 (14)
*Z*	8
Radiation type	Mo *K*α
μ (mm^−1^)	4.60
Crystal size (mm)	0.30 × 0.29 × 0.22

Data collection	
Diffractometer	Bruker D8 Quest
Absorption correction	Multi-scan (*SADABS*; Krause *et al.*, 2015 ▸)
*T*_min_, *T*_max_	0.280, 0.746
No. of measured, independent and observed [*I* > 2σ(*I*)] reflections	3508, 3379, 3094
*R*_int_	0.025
(sin θ/λ)_max_ (Å^−1^)	0.667

Refinement	
*R*[*F*^2^ > 2σ(*F*^2^)], *wR*(*F*^2^), *S*	0.048, 0.079, 1.25
No. of reflections	3379
No. of parameters	158
H-atom treatment	H atoms treated by a mixture of independent and constrained refinement
Δρ_max_, Δρ_min_ (e Å^−3^)	0.96, −1.04
Absolute structure	Refined as an inversion twin
Absolute structure parameter	0.44 (9)

Computer programs: *APEX2* and *SAINT* (Bruker, 2014 ▸), *SHELXS97* (Sheldrick 2008 ▸), *ORTEP-3 for Windows* (Farrugia, 2012 ▸), *Mercury* (Macrae *et al.*, 2020 ▸), *SHELXL2019/3* (Sheldrick, 2015 ▸) and *PLATON* (Spek, 2020 ▸).

## full crystallographic data

### 3-Iodoaniline Crystal data

**Table:**

C_6_H_6_IN	*D*_x_ = 2.140 Mg m^−^^3^
*M**_r_* = 219.02	Mo *K*α radiation, λ = 0.71073 Å
Orthorhombic, *P*2_1_2_1_2_1_	Cell parameters from 9179 reflections
*a* = 5.0748 (3) Å	θ = 2.5–28.3°
*b* = 12.9872 (8) Å	µ = 4.60 mm^−^^1^
*c* = 20.6243 (12) Å	*T* = 200 K
*V* = 1359.29 (14) Å^3^	Block, colourless
*Z* = 8	0.30 × 0.29 × 0.22 mm
*F*(000) = 816	

### 3-Iodoaniline Data collection

**Table:**

Bruker D8 Quest diffractometer	3094 reflections with *I* > 2σ(*I*)
φ and ω scans	*R*_int_ = 0.025
Absorption correction: multi-scan (SADABS; Krause *et al.*, 2015)	θ_max_ = 28.3°, θ_min_ = 2.5°
*T*_min_ = 0.280, *T*_max_ = 0.746	*h* = −6→6
3508 measured reflections	*k* = 0→17
3379 independent reflections	*l* = 0→27

### 3-Iodoaniline Refinement

**Table:**

Refinement on *F*^2^	Secondary atom site location: difference Fourier map
Least-squares matrix: full	Hydrogen site location: mixed
*R*[*F*^2^ > 2σ(*F*^2^)] = 0.048	H atoms treated by a mixture of independent and constrained refinement
*wR*(*F*^2^) = 0.079	*w* = 1/[σ^2^(*F*_o_^2^) + 10.4557*P*] where *P* = (*F*_o_^2^ + 2*F*_c_^2^)/3
*S* = 1.25	(Δ/σ)_max_ < 0.001
3379 reflections	Δρ_max_ = 0.96 e Å^−^^3^
158 parameters	Δρ_min_ = −1.04 e Å^−^^3^
0 restraints	Absolute structure: Refined as an inversion twin
Primary atom site location: structure-invariant direct methods	Absolute structure parameter: 0.44 (9)

### 3-Iodoaniline Special details

**Table:**

Geometry. All esds (except the esd in the dihedral angle between two l.s. planes) are estimated using the full covariance matrix. The cell esds are taken into account individually in the estimation of esds in distances, angles and torsion angles; correlations between esds in cell parameters are only used when they are defined by crystal symmetry. An approximate (isotropic) treatment of cell esds is used for estimating esds involving l.s. planes.
Refinement. Refined as a 2-component inversion twin. The aromatic carbon-bound H atoms were placed in calculated positions (C–H 0.95 Å) and were included in the refinement in the riding model approximation, with *U*(H) set to 1.2*U*_eq_(C). The N–H bonded H atoms were located on a DFM and included in the refinement with with *U*(H) set to 1.5*U*_eq_(N).

### 3-Iodoaniline Fractional atomic coordinates and isotropic or equivalent isotropic displacement parameters (Å^2^)

**Table:**

	*x*	*y*	*z*	*U*_iso_*/*U*_eq_	
I1	−0.29954 (15)	0.23185 (5)	0.36694 (4)	0.03054 (16)	
I2	0.19029 (13)	1.01208 (5)	0.36477 (4)	0.03011 (16)	
N1	0.311 (2)	0.5517 (8)	0.2859 (5)	0.038 (2)	
H711	0.45 (3)	0.581 (12)	0.283 (7)	0.057*	
H712	0.29 (3)	0.523 (10)	0.246 (6)	0.057*	
N2	0.811 (2)	0.6961 (7)	0.2824 (5)	0.035 (2)	
H721	0.79 (3)	0.718 (10)	0.244 (6)	0.053*	
H722	0.95 (3)	0.660 (12)	0.288 (7)	0.053*	
C11	0.246 (2)	0.4856 (8)	0.3367 (5)	0.034 (3)	
C12	0.055 (2)	0.4098 (8)	0.3286 (5)	0.027 (2)	
H12	−0.025586	0.399800	0.287462	0.033*	
C13	−0.017 (2)	0.3494 (8)	0.3806 (5)	0.028 (2)	
C14	0.096 (2)	0.3604 (10)	0.4415 (5)	0.037 (3)	
H14	0.044405	0.317533	0.476599	0.044*	
C15	0.285 (3)	0.4353 (9)	0.4494 (5)	0.040 (3)	
H15	0.365993	0.444220	0.490503	0.048*	
C16	0.359 (2)	0.4986 (9)	0.3974 (5)	0.035 (3)	
H16	0.487506	0.550785	0.403765	0.042*	
C21	0.7402 (19)	0.7588 (8)	0.3351 (5)	0.029 (2)	
C22	0.548 (2)	0.8356 (8)	0.3249 (5)	0.027 (2)	
H22	0.474817	0.847272	0.283107	0.032*	
C23	0.4677 (17)	0.8935 (7)	0.3780 (5)	0.023 (2)	
C24	0.568 (2)	0.8779 (8)	0.4395 (5)	0.032 (2)	
H24	0.507738	0.917882	0.475135	0.038*	
C25	0.757 (2)	0.8032 (8)	0.4478 (5)	0.035 (3)	
H25	0.827830	0.792069	0.489853	0.042*	
C26	0.846 (2)	0.7436 (9)	0.3964 (5)	0.032 (3)	
H26	0.977570	0.692867	0.403175	0.039*	

### 3-Iodoaniline Atomic displacement parameters (Å^2^)

**Table:**

	*U*^11^	*U*^22^	*U*^33^	*U*^12^	*U*^13^	*U*^23^
I1	0.0283 (3)	0.0277 (3)	0.0356 (3)	0.0017 (3)	0.0044 (4)	0.0022 (3)
I2	0.0273 (3)	0.0253 (3)	0.0377 (3)	0.0014 (3)	0.0022 (4)	0.0021 (3)
N1	0.029 (5)	0.036 (5)	0.049 (6)	−0.011 (5)	0.004 (5)	0.004 (4)
N2	0.043 (6)	0.026 (5)	0.037 (5)	0.002 (5)	0.004 (5)	−0.004 (4)
C11	0.027 (7)	0.026 (5)	0.049 (6)	0.004 (5)	−0.005 (5)	−0.005 (5)
C12	0.029 (6)	0.032 (6)	0.021 (5)	0.003 (5)	−0.003 (4)	−0.001 (4)
C13	0.025 (5)	0.028 (5)	0.032 (6)	0.001 (4)	−0.002 (4)	−0.001 (4)
C14	0.038 (7)	0.046 (7)	0.027 (5)	0.007 (6)	0.002 (5)	0.002 (5)
C15	0.041 (7)	0.048 (7)	0.031 (5)	0.007 (6)	−0.012 (5)	−0.010 (5)
C16	0.030 (6)	0.032 (6)	0.043 (6)	−0.014 (5)	0.006 (5)	−0.015 (5)
C21	0.015 (6)	0.036 (6)	0.036 (5)	−0.013 (5)	−0.002 (4)	0.006 (4)
C22	0.025 (6)	0.028 (5)	0.027 (5)	−0.002 (5)	−0.010 (4)	0.006 (4)
C23	0.012 (4)	0.023 (5)	0.033 (5)	−0.004 (4)	0.005 (4)	0.002 (4)
C24	0.038 (6)	0.032 (6)	0.024 (5)	0.000 (5)	0.005 (5)	−0.001 (4)
C25	0.040 (8)	0.038 (6)	0.028 (5)	−0.002 (5)	0.000 (5)	0.004 (4)
C26	0.023 (6)	0.036 (6)	0.037 (5)	−0.003 (5)	−0.005 (5)	0.013 (5)

### 3-Iodoaniline Geometric parameters (Å, º)

**Table:**

I1—C13	2.113 (11)	C14—H14	0.9500
I2—C23	2.105 (10)	C15—C16	1.403 (16)
N1—C11	1.394 (14)	C15—H15	0.9500
N1—H711	0.78 (15)	C16—H16	0.9500
N1—H712	0.92 (13)	C21—C26	1.388 (13)
N2—C21	1.404 (14)	C21—C22	1.412 (15)
N2—H721	0.84 (13)	C22—C23	1.388 (14)
N2—H722	0.84 (15)	C22—H22	0.9500
C11—C16	1.388 (14)	C23—C24	1.381 (14)
C11—C12	1.391 (15)	C24—C25	1.374 (16)
C12—C13	1.378 (14)	C24—H24	0.9500
C12—H12	0.9500	C25—C26	1.389 (15)
C13—C14	1.388 (15)	C25—H25	0.9500
C14—C15	1.375 (17)	C26—H26	0.9500
			
C11—N1—H711	124 (10)	C11—C16—C15	120.5 (10)
C11—N1—H712	113 (8)	C11—C16—H16	119.8
H711—N1—H712	105 (10)	C15—C16—H16	119.8
C21—N2—H721	120 (9)	C26—C21—N2	121.6 (10)
C21—N2—H722	116 (10)	C26—C21—C22	120.2 (10)
H721—N2—H722	114 (10)	N2—C21—C22	118.2 (10)
C16—C11—C12	118.9 (10)	C23—C22—C21	117.9 (9)
C16—C11—N1	120.2 (10)	C23—C22—H22	121.1
C12—C11—N1	120.7 (10)	C21—C22—H22	121.1
C13—C12—C11	119.6 (10)	C24—C23—C22	122.5 (10)
C13—C12—H12	120.2	C24—C23—I2	118.2 (8)
C11—C12—H12	120.2	C22—C23—I2	119.3 (7)
C12—C13—C14	122.4 (11)	C25—C24—C23	118.3 (10)
C12—C13—I1	119.2 (8)	C25—C24—H24	120.8
C14—C13—I1	118.3 (8)	C23—C24—H24	120.8
C15—C14—C13	117.9 (11)	C24—C25—C26	121.6 (10)
C15—C14—H14	121.1	C24—C25—H25	119.2
C13—C14—H14	121.1	C26—C25—H25	119.2
C14—C15—C16	120.7 (10)	C21—C26—C25	119.5 (10)
C14—C15—H15	119.6	C21—C26—H26	120.3
C16—C15—H15	119.6	C25—C26—H26	120.3
			
C16—C11—C12—C13	−0.2 (16)	C26—C21—C22—C23	−0.6 (15)
N1—C11—C12—C13	−176.3 (10)	N2—C21—C22—C23	176.5 (10)
C11—C12—C13—C14	−0.6 (17)	C21—C22—C23—C24	−0.5 (15)
C11—C12—C13—I1	−178.3 (8)	C21—C22—C23—I2	178.0 (7)
C12—C13—C14—C15	0.5 (17)	C22—C23—C24—C25	1.0 (16)
I1—C13—C14—C15	178.2 (8)	I2—C23—C24—C25	−177.5 (8)
C13—C14—C15—C16	0.3 (17)	C23—C24—C25—C26	−0.4 (17)
C12—C11—C16—C15	1.0 (17)	N2—C21—C26—C25	−175.8 (10)
N1—C11—C16—C15	177.1 (11)	C22—C21—C26—C25	1.1 (16)
C14—C15—C16—C11	−1.1 (18)	C24—C25—C26—C21	−0.7 (17)

### 3-Iodoaniline Hydrogen-bond geometry (Å, º)

**Table:**

*D*—H···*A*	*D*—H	H···*A*	*D*···*A*	*D*—H···*A*
N1—H711···N2	0.78 (15)	2.38 (16)	3.156 (17)	170 (15)
N2—H722···N1^i^	0.84 (15)	2.33 (15)	3.157 (18)	171 (13)
C13—I1···I2^ii^	2.11 (1)	3.85 (1)	8.677 (10)	173 (1)
C23—I2···I1^iii^	2.11 (1)	3.79 (1)	9.437 (9)	172 (1)

Symmetry codes: (i) *x*+1, *y*, *z*; (ii) *x*−1, *y*, *z*; (iii) *x*, *y*+1, *z*.
